# Contribution of major histocompatibility complex class II immunostaining in distinguishing idiopathic inflammatory myopathy subgroups: A histopathological cohort study

**DOI:** 10.1093/jnen/nlae098

**Published:** 2024-09-16

**Authors:** Lola E R Lessard, Marie Robert, Tanguy Fenouil, Rémi Mounier, Véréna Landel, Marie Carlesimo, Arnaud Hot, Bénédicte Chazaud, Thomas Laumonier, Nathalie Streichenberger, Laure Gallay

**Affiliations:** Service d’Electroneuromyographie et de pathologies neuromusculaires, Hôpital Neurologique, GHE, Hospices Civils de Lyon, Lyon, France; Institut NeuroMyoGène, Unité Physiopathologie et Génétique du Neurone et du Muscle, CNRS UMR 5261, Inserm U1315, Université Claude Bernard Lyon 1, Lyon, France; Service de Médecine interne et immunologie clinique, Centre Hospitalier Universitaire Édouard Herriot, Hospices Civils de Lyon, Lyon, France; Institut de Pathologie Multisite des Hospices Civils de Lyon-Site Est, GHE, Hospices Civils de Lyon, Lyon, France; Centre de Recherche en Cancérologie de Lyon, Inserm U1052, CNRS UMR 5286, Lyon, France; Institut NeuroMyoGène, Unité Physiopathologie et Génétique du Neurone et du Muscle, CNRS UMR 5261, Inserm U1315, Université Claude Bernard Lyon 1, Lyon, France; Direction de la Recherche en Santé, Hospices Civils de Lyon, Lyon, France; Institut de Pathologie Multisite des Hospices Civils de Lyon-Site Est, GHE, Hospices Civils de Lyon, Lyon, France; Service de Médecine interne et immunologie clinique, Centre Hospitalier Universitaire Édouard Herriot, Hospices Civils de Lyon, Lyon, France; Institut NeuroMyoGène, Unité Physiopathologie et Génétique du Neurone et du Muscle, CNRS UMR 5261, Inserm U1315, Université Claude Bernard Lyon 1, Lyon, France; Laboratoire “Cell Therapy & Musculoskeletal Disorders”, Département de Chirurgie Orthopédique, Hôpital Universitaire et Faculté de Médecine, Genève, Switzerland; Institut NeuroMyoGène, Unité Physiopathologie et Génétique du Neurone et du Muscle, CNRS UMR 5261, Inserm U1315, Université Claude Bernard Lyon 1, Lyon, France; Institut de Pathologie Multisite des Hospices Civils de Lyon-Site Est, GHE, Hospices Civils de Lyon, Lyon, France; Service de Médecine interne et immunologie clinique, Centre Hospitalier Universitaire Édouard Herriot, Hospices Civils de Lyon, Lyon, France; Laboratoire “Cell Therapy & Musculoskeletal Disorders”, Département de Chirurgie Orthopédique, Hôpital Universitaire et Faculté de Médecine, Genève, Switzerland

**Keywords:** idiopathic inflammatory myopathies, major histocompatibility complex-II, muscle biopsy, myofiber, capillary

## Abstract

Idiopathic inflammatory myopathies (IIM) are rare, acquired muscle diseases; their diagnosis of is based on clinical, serological, and histological criteria. MHC-I-positive immunostaining, although non-specific, is used as a marker for IIM diagnosis; however, the significance of major histocompatibility complex (MHC)-II immunostaining in IIM remains debated. We investigated patterns of MHC-II immunostaining in myofibers and capillaries in muscle biopsies from 103 patients with dermatomyositis ([DM], *n* = 31), inclusion body myositis ([IBM], *n* = 24), anti-synthetase syndrome ([ASyS], *n* = 10), immune-mediated necrotizing myopathy ([IMNM], *n* = 18), or overlap myositis ([OM], *n* = 20). MHC-II immunostaining of myofibers was abnormal in 63/103 of patients (61%) but the patterns differed according to the IIM subgroup. They were diffuse in IBM (96%), negative in IMNM (83%), perifascicular in ASyS (70%), negative (61%) or perifascicular (32%) in DM, and either clustered (40%), perifascicular (30%), or diffuse heterogeneous (15%) in OM. Capillary MHC-II immunostaining also identified quantitative (capillary dropout, *n* = 47/88, 53%) and qualitative abnormalities, that is, architectural abnormalities, including dilated and leaky capillaries, (*n* = 79/98, 81%) in all IIM subgroups. Thus, MHC-II myofiber expression patterns allow distinguishing among IIM subgroups. We suggest the addition of MHC-II immunostaining to routine histological panels for IIM diagnosis.

## Introduction

Idiopathic inflammatory myopathies (IIM) are rare acquired muscle disorders, the prevalence of which has been estimated at 14/100 000 individuals.^1^ In the last decade, an increasing understanding of IIM has allowed the identification of five main clinico-pathological subgroups, that is, dermatomyositis (DM), inclusion body myositis (IBM), immuno-mediated necrotizing myopathy (IMNM), anti-synthetase syndrome (ASyS), and overlap myositis (OM).^2–7^ These IIM subgroups differ in terms of clinical features, muscle biopsy findings and detected auto-antibodies, notably myositis associated antibodies (MSA). In clinical practice, the final diagnosis of IIM is sometimes difficult, which can lead to a significant delay in patient care.^8^ Because therapeutic approaches greatly vary among the different IIM subgroups, readily available tools that can help distinguish between these subgroups, and thus reduce diagnostic delays, are warranted to improve patient management.

The major histocompatibility complex (MHC) corresponds to cell surface glycoproteins entrusted with antigen presentation. The two major classes of MHC, MHC-I and MHC-II molecules, present antigenic peptides to CD8+ T cells and CD4+ T cells, respectively.^9^ While all nucleated cells express MHC-I, MHC-II is normally only expressed by “professional” antigen-presenting cells (APCs) such as B cells, monocytes, macrophages, and dendritic cells, as well as several other cell types upon inflammatory signals, notably epithelial cells.^10^ However, histological analyses show that MHC-I and MHC-II are not detected on healthy and mature human skeletal myofibers, while both MHC-I and MHC-II are detected on endothelial cells, thta is, capillaries, presenting a punctiform-positive immunostaining.^11–13^ Myofiber expression of MHC-I, also known as HLA-ABC, is used as a diagnostic tool for IIM. However, given its high sensitivity but low specificity, additional clinical and pathological elements are required to reach the diagnosis.^14–19^ Few studies have investigated the expression of MHC-II (also known as HLA-DR/HLA-DQ/HLA-DP) in IIM muscles.^12^^,^^14–18^^,^^20–34^ Among these, several studies have demonstrated that, while MHC-I positivity is frequently observed in both IIM and hereditary myopathies, MHC-II immunostaining can distinguish IIM from hereditary myopathies.^12^^,^^15^^,^^17^^,^^18^^,^^20^^,^^23^^,^^25^^,^^26^^,^^28^^,^^30^^,^^31^^,^^35^ More specifically, the study by Rodriguez et al, which included 186 IIM and 246 cases of non-inflammatory myopathies (including 200 hereditary myopathies), found that MHC-II was positive in 61.7% of IIM but in only 10.1% of non-inflammatory cases.^18^ These data highlight the sensitivity of MHC-I immunostaining and the specificity of MHC-II immunostaining. Moreover, to our knowledge, except for 4 studies specific to certain IIM subgroups (DM, juvenile DM, ASyS),^21^^,^^32–34^ most studies were conducted 10-40 years ago, and thus did not take into account the current IIM classification. Based on these data, recent expert opinion (EURO-NMD pathology working group) recommended that MHC-II immunostaining of muscle biopsy remains optional for the diagnosis of IIM.^36^

The present study aimed to evaluate the usefulness of MCH-II immunostaining in the diagnosis of IIM. To that end, the patterns of myofiber and capillary MHC-II immunostaining were investigated in muscle biopsies of patients with DM, IBM, IMNM, ASyS, and OM.

## Methods

### Patients

A historical cohort was created using the MYOLYON registry, an IIM database of the Lyon University Hospital (France), by screening for patients diagnosed with IIM between 2017 and 2023. The inclusion criteria were set up in accordance with the current international IIM diagnosis criteria, based on clinical, pathological, and serological data: DM, IBM,^2^^,^^37^ ASyS,^5^^,^^6^^,^^38^ IMNM,^3^ or OM^6^^,^^7^^,^^39^ (Table 1). Exclusion criteria were differential diagnoses (mainly hereditary myopathy, toxic or iatrogenic myopathy, and infectious diseases) and the unavailability of medical records.

**Table 1.** Diagnostic criteria for idiopathic inflammatory myopathies.

Diagnosis	Clinical criteria	Pathological criteria	Serological criteria	Ref
**DM**	DM clinical features along with either DM muscle features or a DM-specific autoantibody (classic DM) OR DM muscle features and DM-specific autoantibody (DM *sine* dermatitis). DM clinical features: at least two of the following: Gottron’s sign, Gottron’s papules (and equivalent as reported in (31)), and/or heliotrope rash.	DM muscle features: (a) 1 and 2, or (b) 1 and 3, or (c) 2 and 3, or (d) 4. 1. Proximal muscle weakness. 2. Elevated muscle enzymes. 3. Suggestive DM muscle biopsy findings: lymphocytic infiltrate (perivascular), evidence of perifascicular disease (perifascicular predominant fibers that are pale on COX staining and/or positive on NCAM staining). 4. Definitive DM muscle biopsy findings: perifascicular atrophy and/or perifascicular MxA overexpression with rare or absent perifascicular necrosis.	DM-specific autoantibodies (TIF1γ, NXP2, Mi2, MDA5, SAE).	(4)
**IBM**	All four criteria: 1. Duration >12 months. 2. Age at onset >45 years. 3. Finger flexor and/or quadriceps weakness. 4. Muscle enzymes no greater than 15× ULN.	Both criteria: 1. Endomysial inflammation. 2. Either invasion of nonnecrotic muscle fibers or rimmed vacuoles or up-regulation of MHC-I.	None.	(2, 37)
**Anti-HMGCR IMNM**	Both criteria: 1. Proximal muscle weakness. 2. Elevated muscle enzymes.	None.	Anti-HMGCR autoantibodies.	(3)
**Anti-SRP IMNM**	Both criteria: 1. Proximal muscle weakness. 2. Elevated muscle enzymes.	None.	Anti-SRP autoantibodies.	(3)
**Antibody negative IMNM**	Both criteria: 1. Proximal muscle weakness. 2. Elevated muscle enzymes.	All three criteria: 1. Scattered necrotic myofibers. 2. Different stages of necrosis, myophagocytosis and regeneration. 3. Macrophage predominant, paucilymphocytic infiltrates.	Absence of myositis-specific autoantibodies.	(3)
**ASyS**	At least one among the following: 1. Myositis. 2. Interstitial lung disease. 3. Arthritis. 4. Raynaud’s phenomenon. 5. Mechanic’s hands.	At least one among the following: 1. Perifascicular necrotic fibers. 2. Inflammation from perimysium to endomysium. 3. Perifascicular MHC-I and MHC-II. 4. Sarcolemmal complement deposition in the perimysium.	Anti-aminoacyl-RNA-synthetase autoantibodies (Jo1, PL7, PL12, EJ, OJ, KS, Zo, HA/YRS).	(5, 6, 38)
**OM**	Inflammatory myopathy, at least two among the following: 1. Muscle weakness. 2. Elevated muscle enzymes. 3. Myopathic electromyogram. Connective tissue disease or symptoms of connective tissue disease (including cutaneous, articular or lung involvement)	All three criteria: 1. Perimysial and perivascular localization of mononuclear cells. 2. Without additional endomysial infiltrate. 3. Without perifascicular atrophy.	Connective tissue disease associated autoantibodies (centromeres, topo I, RNA-polymerases I or III, Th; U1RNP, U2RNP, U3RNP, U5RNP, Pm-Scl, Ku, nucleoporins).	(6, 7, 39)

The idiopathic inflammatory myopathies diagnosis criteria applied in the present study was defined in accordance with the current international classification. Exclusion criteria were used as defined in cited references for each IIM subgroup.

Abbreviations: ASyS = anti-synthetase syndrome; DM = dermatomyositis. HMGCR = hydroxyméthylglutaryl-CoA reductase; IBM = inclusion body myositis; IMNM = immune-mediated necrotizing myopathy; MDA5 = melanoma differentiation gene 5; MHC-I = major histocompatibility class I, MHC-II = major histocompatibility class II, Mi2 = complex nucleosome remodeling histone deacetylase, MxA = myxovirus resistance protein A; NXP-2 = nuclear matrix protein 2; OM = overlap myositis; SAE = small ubiquitin-like modifier-activating enzyme; SRP = signal recognition particle; TIF1 γ = transcriptional intermediary factor 1 gamma; ULN = upper limit normal.

### Histopathology

All the immunostainings were performed using an automated immunohistochemistry instrument (Ventana, Ultra Benchmark, Ventana Medical Systems, Inc., Tucson, AZ, United States) on 7-μm muscle cryosections using the following primary antibodies: anti-MHC-I (clone W6/32, DAKO, ref: M0736, 1/2400), anti-MHC-II (clone CR3/43, DAKO, ref: M0775, 1/400, corresponding to Anti-HLA DR + DP + DQ antibody), anti-CD56 (clone 123C3, Cell Marque, ref: 156-M85, 1/50) Leica Biosystems NCL-CD56-1B6), and anti-CD31 (clone JC70A, Ventana, ref: 760-4378, prediluted, Supplementary Data S1). All primary antibodies were revealed using the ultraView Universal DAB Detection Kit (Ventana, ref: 05269806001). Immunostainings were analyzed by four experts in neuromuscular pathology: two neuromuscular pathologists (N.S., T.F.), a rheumatologist (L.G.), and a neurologist (L.L.); the biopsies were blinded in terms of final diagnosis and any parameter that would allow patient identification. To facilitate the analysis, a healthy control muscle sample was added on the edge of each slide for each staining. Biopsies with questionable or faint staining or a staining fainter than the background were considered as negative. Digital image capture was performed using an Axio Scan.Z1 (Zeiss).

#### Myofiber immunohistochemistry

The positivity of the immunostaining was evaluated in a minimum of three representative fascicles and defined in the most affected fascicle by (1) the presence of positive myofibers in the whole fascicle and (2) the distribution of the positive myofibers. The expression of MHC-II and MHC-I by myofibers was considered positive in the presence of sarcolemmal staining (associated or not with sarcoplasmic staining); only non-necrotic myofiber positivity was considered. The different MHC-I and MHC-II immunostaining patterns are described in Table 2. Immunostainings were evaluated on serial sections of the same sample to evaluate the co-expression of MHC-I, MHC-II, and CD56 by the same myofibers. The co-expression of MHCII and CD56 was investigated in order to characterize the myogenic status of the myofibers that express MHC-II. CD56 positivity was defined by a sarcoplasmic immunostaining of the myofibers.

**Table 2.** Definitions of the various immunostaining patterns.

**Myofiber MHC-II and MHC-I immunostaining patterns**	
Diffuse	
Homogenous	Diffuse and homogenous myofiber positivity in the muscle biopsy
Heterogeneous	Diffuse and heterogeneous myofiber positivity in the muscle biopsy
Perifascicular pattern	
Strictly perifascicular	Positive myofibers only in the perifascicular area, extended to a maximum of 4-cell layers
Extended perifascicular	Diffuse positive immunostaining of myofibers according to a positivity gradient with a maximal intensity in the perifascicular area
Scattered	Positive myofibers scattered throughout the muscle biopsy with at least five myofibers per fascicle
Clustered^a^	Positive immunostaining of myofibers around necrotic myofibers and/or inflammatory infiltrates, including a minimum of ten positive myofibers
Negative	No myofiber positivity
**Capillary MHC-II and CD31 immunostaining patterns**	
Capillary dropout	At least three myofibers with no related capillaries, found at least three times in the muscle biopsy
Leaky	Positive immunostaining of capillaries brimming over capillary edges, giving a blurred aspect
Dilated	Positive immunostaining of oversize capillaries with marked thickening and diameter-increasing in transversal section
Normal	Punctiform immunostaining without visible lumen

a Only found with MHC-II immunostaining.

Abbreviation: MHC = major histocompatibility complex.

#### Capillary immunohistochemistry

Capillaries were evaluated in a minimum of three fascicles on transverse sections. A normal pattern was considered in the presence of at least one capillary per myofiber, which displayed a punctiform immunostaining without visible lumen. Immunostainings were evaluated on serial sections of the same sample to evaluate the co-expression of MHC-II and CD31 (gold standard for capillary endothelial cell immunostaining)^36^ in order to evaluate the density and architecture of the capillaries. The different MHC-II and CD31 immunostaining patterns are described in Table 2.

### Statistical analysis

Descriptive analysis and frequency calculations were performed using Microsoft Excel. Medians were reported with interquartile range (IQR).

## Results

A total of 103 patients with a final diagnosis of IIM and available muscle biopsies were included: 31 DM, 24 IBM, 18 IMNM, 10 ASyS, and 20 OM. The overall median [IQR] age was 57 years old [ie, 49-73] and sex ratio was 0.6. Among the 103 patients, 13 received immunosuppressive drugs before the muscle biopsy (DM *n* = 6, ASyS, IBM, and OM *n* = 2 each, and IMNM *n* = 1). In the DM subgroup, MSA was found in 71% of the cases, which were apportioned as follows: anti-TIF1γ antibody (Ab) *n* = 10/31, anti-NXP2 Ab *n* = 5/31, anti-Mi2 Ab *n* = 3, anti-SAE Ab *n* = 2/31, and anti-MDA5 Ab *n* = 2/3. Regarding the other patients, 12/24 IBM presented anti-Cn1A Ab, 14/18 IMNM had either anti-HMGCR Ab (*n* = 12) or anti-SRP Ab (*n* = 2), ASyS patients all had anti-RNA synthetase Ab (anti-Jo1 *n* = 6, anti-PL7 *n* = 4), and 13/20 OM patients had autoantibodies (mainly anti-SmRNP n = 4, anti-Ku n = 3, anti-Scl70 n = 2; Table 3). Overall, an abnormal MHC-II immunostaining of myofibers was found in 63/103 (61%) patients: 11/31 (35%) DM, 24/24 (100%) IBM, 3/18 (17%) IMNM, 9/10 (90%) ASyS, and 16/18 (80%) OM patients. MHC-II immunostaining also highlighted quantitative capillary abnormalities in 47/88 (53%) patients and qualitative capillary abnormalities in 79/98 (81%) patients.

**Table 3.** Individual diagnostic characteristics of patients with idiopathic inflammatory myopathies according to each subgroup.

Dermatomyositis									
N	Gender/age, years	DM clinical features	DM muscle features	DM specific autoantibody	Diagnosis	MHC-I positivity	MHC-I pattern	MHC-II positivity	MHC-II pattern
1	F/65	Yes	1.4.	TIF1γ	CADM (ovarian adenocarcinoma)	+	Diffuse homo	–	–
2	M/59	Yes	1.2.4.	NXP2	DM	+	Diffuse homo	+	Perifascicular
3	M/5	Yes	1.3.4.	TIF1γ	Juvenile DM	+	Diffuse homo	+	Perifascicular, clusters
4	M/8	Yes	1.2.3.4.	NXP2	Juvenile DM	+	Diffuse homo	+	Perifascicular
5	M/55	Yes	1.2.3.4.	None	DM	+	Diffuse homo	–	–
6	F/73	Yes	1.2.	TIF1γ	CADM (melanoma)	+	Diffuse homo	+	Diffuse
7	M/43	Yes	1.3.4.	None	Seronegative DM	+	Perifascicular	+	Perifascicular
8	M/89	Yes	4.	Mi2	DM	+	Perifascicular	–	–
9	F/74	Yes	1.3.4.	TIF1γ	CADM (bronchial adenocarcinoma)	+	Extended perifascicular	+	Perifascicular, clusters
10	F/57	Yes	1.2.	NXP2	DM	+	Perifascicular	–	–
11	F/63	Yes	3.4.	SAE	CADM (bronchial adenocarcinoma)	+	Extended perifascicular	+	Perifascicular
12	F/60	No	1.3.	MDA5	DM *sine* dermatitis	+	Diffuse homo	–	–
13	M/78	No	1.2.	Mi2	DM *sine* dermatitis	–	–	–	–
14	M/4	No	1.2.4.	NXP2	Juvenile DM *sine* dermatitis	+	Extended perifascicular	–	–
15	F/13	Yes	1.2.3.4.	None	Seronegative juvenile DM	+	Extended perifascicular	–	–
16	F/15	Yes	1.2.3.4.	None	Seronegative juvenile DM	+	Extended perifascicular	–	–
17	F/7	Yes	1.3.4.	TIF1γ	Juvenile DM	+	Extended perifascicular	+	Perifascicular
18	M/8	Yes	1.2.	NXP2	Juvenile DM	+	Diffuse homo	–	–
19	M/35	Yes	2.3.	SAE	DM	+	Extended perifascicular	+	Perifascicular
20	M/72	Yes	1.2.3.	Mi2	DM	+	Perifascicular	–	–
21	M/28	Yes	1.3.	MDA5	DM	+	Diffuse homo	–	–
22	M/71	Yes	1.2.4.	TIF1γ	CADM (melanoma)	+	Extended perifascicular	+	Extended perifascicular
23	F/57	Yes	1.2.3.4.	TIF1γ	CADM (ovarian adenocarcinoma)	+	Diffuse homo	–	–
24	H/82	Yes	1.2.3.4.	TIF1γ	CADM (ear carcinoma)	+	Extended perifascicular	–	–
25	F/65	Yes	1.4.	None	DM	+	Perifascicular	–	–
26	F/63	Yes	2.4.	None	DM	+	Extended perifascicular	+	Perifascicular
27	F/33	Yes	1.2.3.	None	DM	+	Diffuse homo	–	–
28	F/64	Yes	1.2.	None	DM	+	Diffuse homo	–	–
29	F/54	Yes	1.2.3.4.	TIF1γ	DM	+	Diffuse homo	–	–
30	F/68	Yes	1.2.3.	None	DM	+	Perifascicular	–	–
31	F/39	Yes	1.2.	TIF1γ	DM	+	Extended perifascicular	+	Perifascicular


Inclusion body myositis									
N	Gender/age, years	All 4 clinical criteria	Endomysial inflammation	Invasion of nonnecrotic muscle fibers or rimmed vacuoles	Up-regulation of MHC-I	MHC-I positivity	MHC-I pattern	MHC-II positivity	MHC-II pattern
1	F/75	Yes	Yes	Yes	Yes	+	Diffuse homo	+	Clusters
2	F/51	Yes	Yes	Yes	Yes	+	Diffuse hetero	+	Diffuse homo
3	M/69	Yes	Yes	Yes	Yes	+	Diffuse hetero	+	Diffuse homo
4	F/80	Yes	Yes	Yes	Yes	+	Diffuse hetero	+	Diffuse homo
5	F/53	Yes	Yes	Yes	Yes	+	Diffuse hetero	+	Diffuse hetero
6	F79	Yes	Yes	No	Yes	+	Diffuse homo	+	Diffuse hetero
7	M/76	Yes	Yes	Yes	Yes	+	Diffuse hetero	+	Diffuse hetero
8	M/73	Yes	Yes	Yes	Yes	+	Diffuse homo	+	Diffuse hetero, clusters
9	M/62	Yes	Yes	Yes	Yes	+	Diffuse homo	+	Diffuse hetero
10	M/77	Yes	Yes	Yes	Yes	+	Diffuse homo	+	Diffuse hetero, clusters
11	F/87	Yes	Yes	Yes	Yes	+	Diffuse homo	+	Diffuse hetero
12	M/46	Yes	Yes	Yes	Yes	+	Diffuse homo	+	Diffuse hetero
13	M/73	Yes	Yes	Yes	Yes	+	Diffuse homo	+	Diffuse hetero
14	F/70	Yes	Yes	Yes	Yes	+	Diffuse homo	+	Clusters
15	F/49	Yes	Yes	Yes	Yes	+	Diffuse homo	+	Diffuse hetero
16	M/66	Yes	Yes	Yes	Yes	+	Diffuse homo	+	Diffuse homo
17	H/75	Yes	Yes	Yes	Yes	+	Diffuse homo	+	Diffuse hetero
18	F/75	Yes	Yes	Yes	Yes	+	Diffuse homo	+	Diffuse hetero
19	H/61	Yes	Yes	Yes	Yes	+	Diffuse homo	+	Clusters
20	F/68	Yes	Yes	Yes	Yes	+	Diffuse homo	+	Clusters
21	F/80	Yes	Yes	Yes	Yes	+	Diffuse homo	+	Diffuse homo
22	F/78	Yes	Yes	Yes	Yes	+	Diffuse homo	+	Clusters
23	H/50	Yes	Yes	Yes	Yes	+	Diffuse homo	+	Diffuse homo
24	H/70	Yes	Yes	Yes	Yes	+	Diffuse homo	+	Diffuse hetero


Immune-mediated necrotizing myopathy								
N	Gender/age, years	Clinical criteria	Pathological criteria	Serological criteria	MHC-I positivity	MHC-I pattern	MHC-II positivity	MHC-II pattern
1	M/68	Yes	1.2.3.	Absence of myositis-specific autoantibody	+	Scattered	–	–
2	M/63	Yes	1.2.	Anti-HMGCR	–	–	–	–
3	F/50	Yes	1.2.	Anti-SRP	+	Scattered	+	Scattered
4	F/54	Yes	1.2.	Anti-HMGCR	+	Scattered	–	–
5	F/40	Yes	1.2.	Anti-HMGCR	+	Scattered	–	–
6	F/11	Yes	None	Anti-HMGCR	+	Diffuse hetero	–	–
7	M/66	Yes	1.2.	Anti-HMGCR	+	Scattered	+	Scattered
8	F/58	Yes	1.2.	Anti-HMGCR	+	Scattered	–	–
9	M/67	Yes	1.2.	Anti-HMGCR	–	–	–	–
10	F/62	Yes	1.2.	Anti-SRP	+	Diffuse hetero	+	Scattered
11	M/64	Yes	1.2.	Anti-HMGCR	+	Diffuse hetero	–	–
12	M/63	Yes	1.2.	Anti-HMGCR	+	Scattered	–	–
13	M/55	Yes	1.2.	Anti-HMGCR	+	Scattered	–	–
14	M/80	Yes	1.2.3.	Absence of myositis-specific autoantibody	+	Scattered	–	–
15	F/49	Yes	1.2.3.	Absence of myositis-specific autoantibody	+	Scattered	–	–
16	F/80	Yes	1.2.	Anti-HMGCR	+	Scattered	–	–
17	H/81	Yes	1.2.	Anti-HMGCR	+	Scattered	–	–
18	F/68	Yes	1.2.3.	unknown	+	Scattered	–	–


Anti-synthetase syndrome								
N	Gender/age, years	Clinical criteria	Pathological criteria	Serological criteria	MHC-I positivity	MHC-I pattern	MHC-II positivity	MHC-II pattern
1	F/57	3.	3.	PL-7	+	Extended perifascicular	–	–
2	F/30	1.3.	1.3.4.	JO-1	+	Perifascicular	+	Perifascicular
3	F/33	1.	1.2.3.4.	JO-1	+	Perifascicular	+	Perifascicular, clusters
4	M/89	1.2.3.4.5.	2.	PL-7	+	Clustered	+	Clusters
5	F/62	1.2.	4.	PL7	+	Diffuse homo	+	Extended perifascicular
6	M/66	2.3.	1.3.4.	JO-1	+	Extended perifascicular	+	Perifascicular
7	F/55	1.2.3.	1.2.3.4.	JO-1	+	Perifascicular	+	Perifascicular
8	F/12	3.	1.2.3.4.	JO-1	+	Perifascicular	+	Perifascicular
9	F/44	2.	2.	OJ PL-7	+	Clustered	+	Scattered
10	F/24	1.3.	1.2.4.	JO-1	+	Diffuse homo	+	Diffuse homo

Overlap myositis									
N	Gender/age, years	Clinical criteria	Connective tissue disease	Pathological criteria	Serological criteria	MHC-I positivity	MHC-I pattern	MHC-II positivity	MHC-II pattern
1	M/21	1.2.	CTD	Yes	Anti-SmRNP, -SSA, -SSB	+	Diffuse homo	+	Scattered
2	F/47	1.2.	CTD	Yes	Anti-nuclear antibodies (speckled)	+	Diffuse homo	+	Diffuse hetero
3	F/54	1.2.	Scleromyositis	Yes	Anti-fibrillarine	+	Extended perifascicular	–	–
4	M/39-77	1.2.3.	Scleromyositis	Yes	Anti-Scl70	–	–	–	–
5	F/70	1.2.	CTD	Yes	Anti-Ku	+	Diffuse homo	+	Clusters, scattered
6	F/74	1.2.	CTD/Sjögren S.	Yes	Anti-centromeres, -SSA, -SSB	+	Diffuse hetero	+	Clusters
7	F/64	1.3.	CTD/RA/Sjögren S.	Yes	Anti-dsDNA, -chromatine, -SSA, -SSB	+	Extended perifascicular	–	–
8	F/70	1.2.	CTD/Sjögren S.	Yes	Anti-SmRNP, -SSA	+	Diffuse homo	+	Clusters
9	F/63	2.	Scleromyositis	Yes	Anti-Ku	+	Diffuse homo	+	Diffuse hetero
10	M/48	1.2.	Sharp syndrome	Yes	Anti-SmRNP, U1RNP	+	Extended perifascicular	+	Perifascicular
11	F/32	1.2.	CTD/scleromyositis	Yes	Anti-Sm, -SmRNP, -U1RNP, -chromatine	+	Perisfascicular	+	Perifascicular
12	F/55	1.2.3.	Scleromyositis	Yes	Anti-Slc70	+	Perisfascicular	+	Perifascicular, clusters
13	F/51	1.	CTD		Anti-mitochondria	+	Diffuse homo	+	Clusters
14	F/77	1.	Scleromyositis		Anti-Pm/Scl	+	Diffuse homo	+	Perifascicular
15	F/82	1.	CTD		Anti-RNA polymerase III	+	Scattered	+	Clusters
16	F/81	1.	CTD		Anti-titine	+	Diffuse hetero	+	Clusters
17	F/71	1.	CTD		Anti-nuclear antibodies	+	Extended perifascicular	+	Perifascicular
18	F/42	1.	CTD		Anti-Ku	–	–	–	–
19	F/71	1.	Scleromyositis		Anti-nuclear antibodies	+	Perisfascicular	+	Extended perifascicular
20	F/74	1.	Scleromyositis		Anti-nuclear antibodies	+	Extended perifascicular	+	Clusters

The ages indicated are the ages of the patient at the time of the muscle biopsy.

Abbreviations: CADM = cancer-associated dermatomyositis; CTD = connective tissue disease; F = female; M = male.

### Myofiber immunohistochemistry study

#### Abnormal expression of MHC-II, MHC-I, and CD56 in IIM myofibers

**Figure 1.**
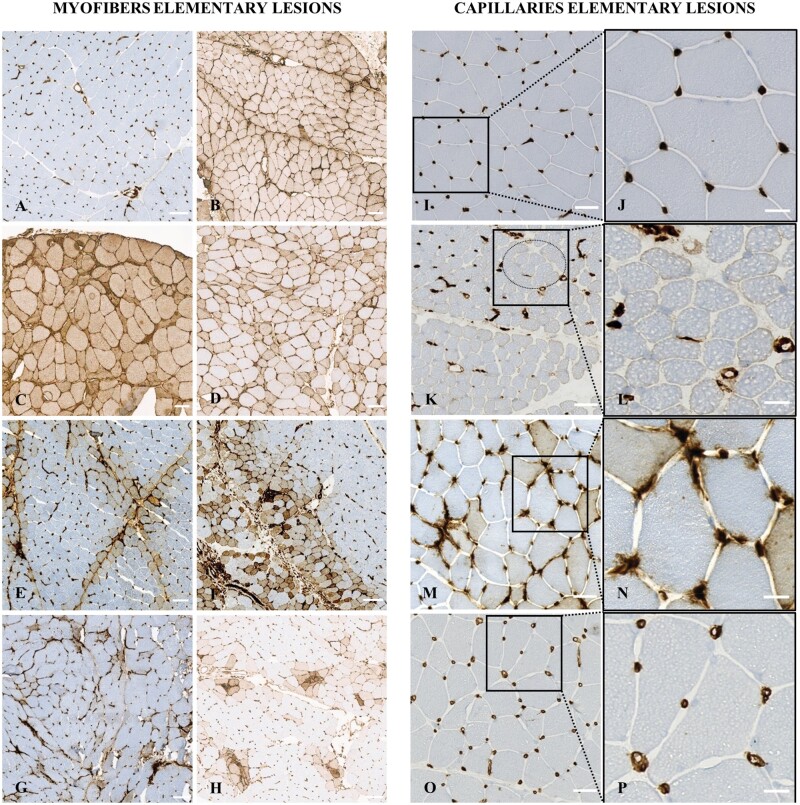
Elementary lesions of myofibers and capillaries observable upon major histocompatibility class II (MHC-II) immunostaining in patients with idiopathic inflammatory myopathies. (A) Normal expression in control muscle biopsy. (B) Diffuse homogenous myofiber positivity in a patient with inclusion body myositis. (C, D) Diffuse heterogeneous myofiber positivity in a patient with inclusion body myositis and overlap myositis, respectively. (E) Strictly perifascicular myofiber positivity in a patient with anti-synthetase syndrome. (F) Extended perifascicular myofiber positivity immunostaining in a patient with dermatomyositis. (G) Scattered myofiber positivity in a patient with immune mediated necrotizing myopathy. (H) Clustered myofiber positivity in a patient with overlap myositis. (I, J) Normal MHC-II expression on capillaries at low magnification (I) and inset of high magnification (J) in a control patient. (K, L) Capillary dropout (dotted ellipse) at low magnification (K) and inset of high magnification (L) in a patient with juvenile dermatomyositis. M-P: Leaky capillaries in a patient with overlap myositis (M, N). Dilated capillaries in a patient with immune mediated necrotizing myopathy (O, P). Low magnifications (M, O) and insets high magnifications (N, P) observable upon MHC-II immunostaining. Scale bars: A-H = 100 μm, I, K, M, O = 50 μm; J, L, N, P = 10 μm.

Myofiber MHC-II immunostaining exhibited five different patterns: negative, diffuse, perifascicular, scattered, or clustered (Table 2; Figure 1). Diffuse positive immunostaining was either homogenous or heterogenous. Perifascicular immunostaining was either strictly perifascicular or extended perifascicular. Among the 63 patients who presented with MHC-II myofiber-positive immunostaining, the variations in MHC-II myofiber patterns were as follows: 38% negative (*n* = 39/103), 8% diffuse homogenous (*n* = 8/103), 19% diffuse heterogeneous (*n* = 20/103), 18% strictly perifascicular (*n* = 19/103), 4% extended perifascicular (*n* = 4/103), 8% scattered (*n* = 8/103), and 12% clustered (*n* = 12/103). Of note, seven cases presented two different patterns: perifascicular and clustered positivity (*n* = 4), clustered and diffuse heterogeneous positivity (*n* = 2), and clustered and scattered positivity (*n* = 1; Supplementary Data S2).

**Figure 2.**
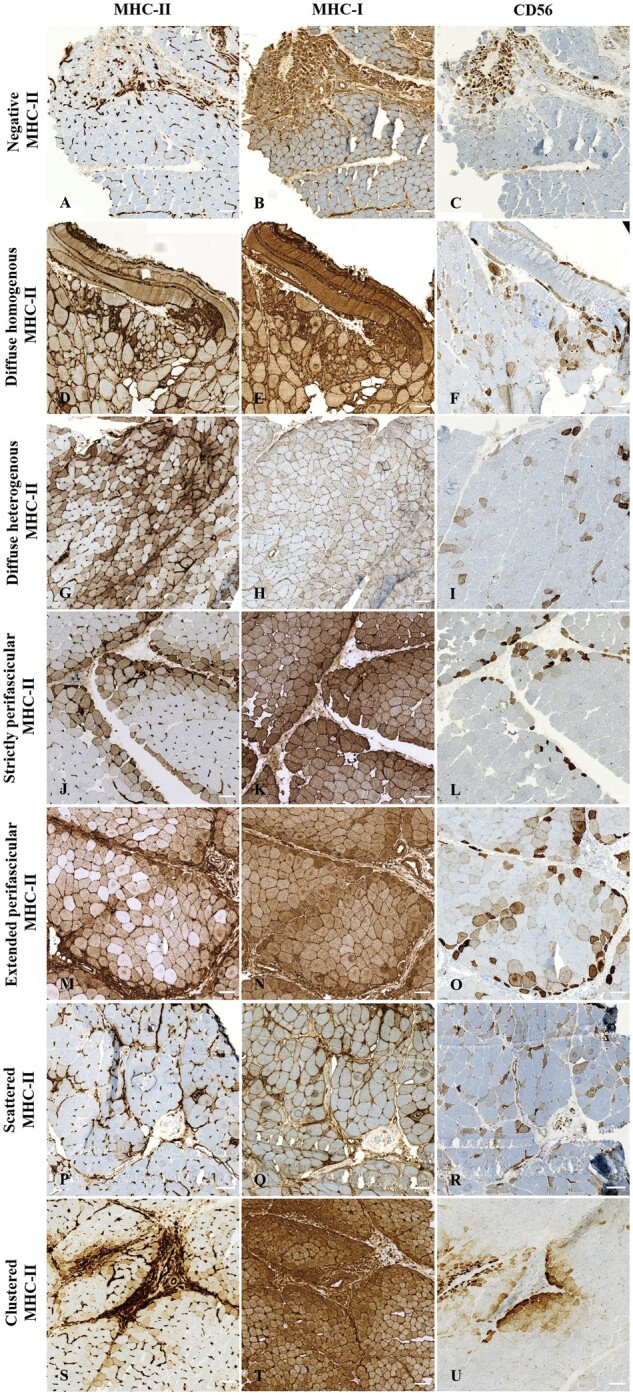
Illustrative images of the different patterns of major histocompatibility complex (MHC) class II, MHC-I, and CD56 myofiber immunostaining on serial sections of muscle biopsies in idiopathic inflammatory myopathies. Left panel shows MHC-II immunostaining, middle panel shows MHCI-I immunostaining, and right panel shows CD56 immunostaining on serial sections of 7 IIM patients. (A-C) Negative MHC-II myofiber immunostaining (A) and corresponding diffuse MHC-I (B) and CD56 (C) immunostaining in a patient with juvenile dermatomyositis. (D-F) Diffuse homogenous MHC-II myofiber positivity (D) and corresponding diffuse MHC-I (E) and CD56 (F) immunostaining in a patient with inclusion body myopathy. (G-I) Diffuse heterogeneous MHC-II myofiber positivity (G) and corresponding diffuse MHC-I (H) and CD56 (I) immunostaining in a patient with inclusion body myopathy. (J-L) Strictly perifascicular MHC-II myofiber positivity (J) and corresponding diffuse MHC-I (K) and CD56 (L) immunostaining in a patient with anti-synthetase syndrome. (M-O) Extended perifascicular MHC-II myofiber positivity (M) and corresponding diffuse MHC-I (N) and CD56 (O) immunostaining in a patient with anti-synthetase syndrome. (P-R) Scattered MHC-II myofiber positivity (P) and corresponding diffuse MHC-I (Q) and CD56 (R) immunostaining in a patient with overlap myositis. (S-U) Clustered MHC-II myofiber positivity (S) and corresponding diffuse MHC-I (T) and CD56 (U) immunostaining in a patient with juvenile dermatomyositis. Scale bars = 100 μm.

MHC-I expression by myofibers was observed in 98/103 (95%) IIM patients. MHC-I staining exhibited the same types of patterns except for the positive clustered myofiber pattern, which was not found (Table 2). The various MHC-I-positive myofiber patterns were found as follows: 5% negative (*n* = 5/103), 38% diffuse homogenous (*n* = 39/103), 12% diffuse heterogeneous (*n* = 12/103), 11% strictly perifascicular (*n* = 11/103), 19% extended perifascicular (*n* = 20/103), and 17% scattered (*n* = 17/103; Supplementary Data S2). CD56 was expressed by some IIM myofibers in all but one tested muscle biopsy (*n* = 97/98, 99%; Figure 2; Supplementary Data S2).

#### Differential MHC-II myofiber expression according to IIM subgroup

**Figure 3.**
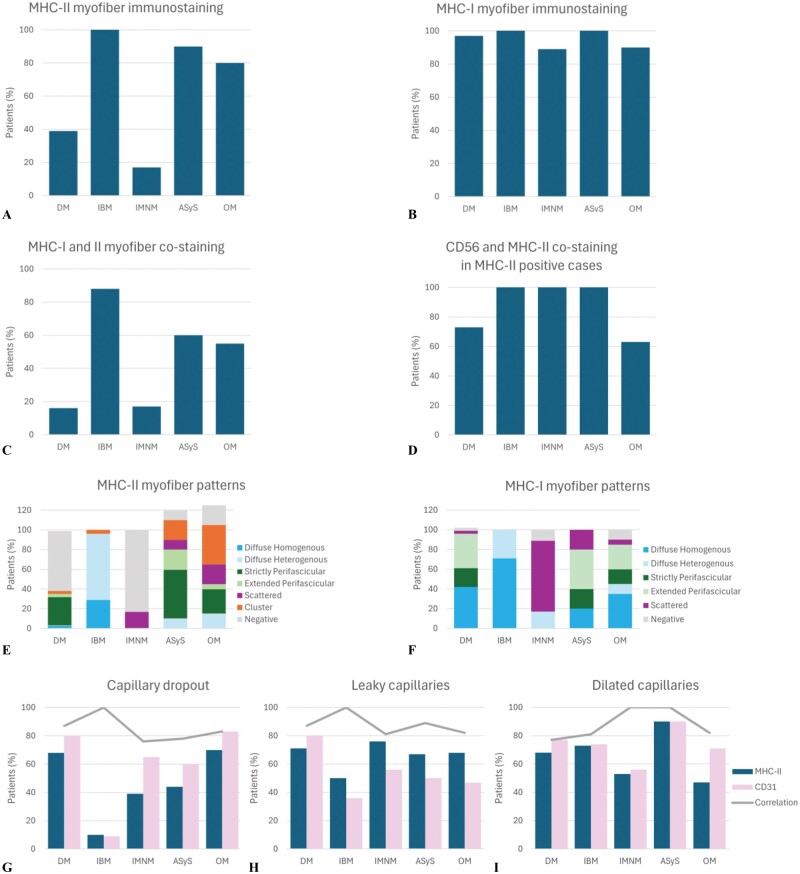
Graphical representation of myofiber and capillary immunostaining results according to idiopathic inflammatory myopathy subgroup. (A) Major histocompatibility complex (MHC) class II. (B) MHC class I myofiber immunostaining in IIM patients according to subgroup. (C) MHC-I and II patterns of expression of myofibers were compared and classified either as “co-staining” (similar pattern of expression) or “no- co-staining” (different patterns). (D) CD56 and MHC-II patterns of expression of myofibers were compared and classified either as “co-staining” (CD56-positive myofibers were also MHC-II-positive and/or *vice versa*) or “no- co-staining” (myofibers expressed either CD56 or MHC-II but not the two markers). (E) MHC class II and (F) MHC class I patterns of expression by myofibers in IIM patients according to subgroup. (G) Capillary dropout, (H) leaky, and (I) dilated capillaries assessed by MHC-II and CD31 immunostaining. Correlation was retained when both techniques showed the same pattern of capillary morphological abnormality.

**Figure 4.**
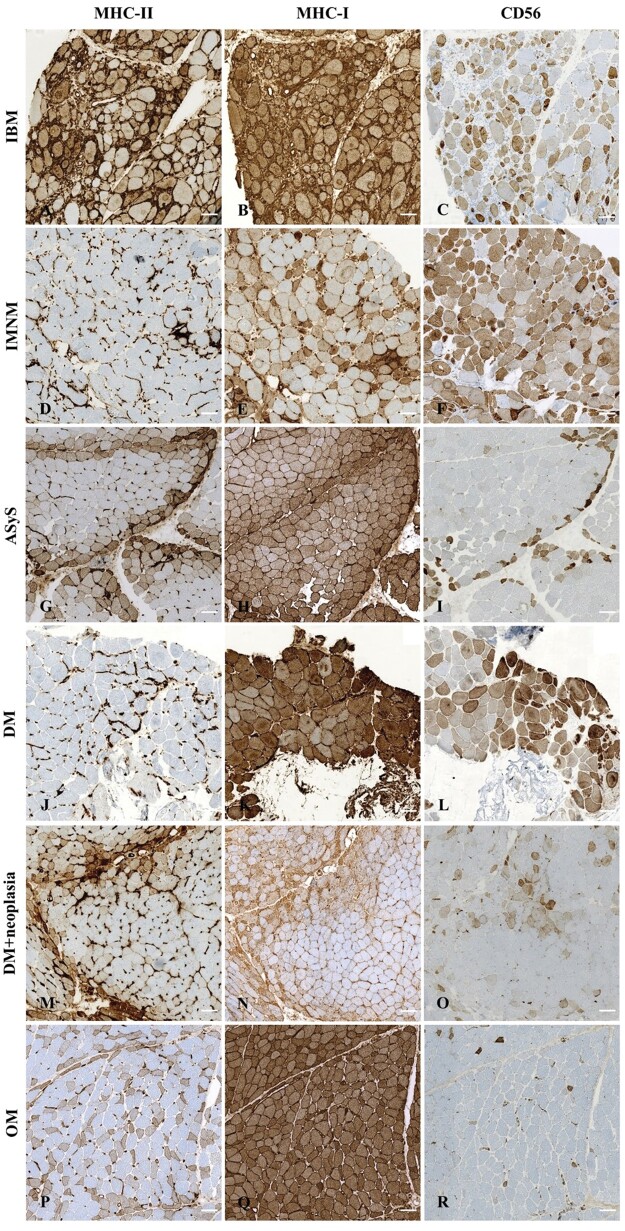
Illustrative images of major histocompatibility complex (MHC) class II, MHC-I, and CD56 myofiber immunostaining in each idiopathic inflammatory myopathy subgroup. Left panel shows MHC-II immunostaining, middle panel shows MHCI-I immunostaining, and right panel shows CD56 immunostaining on serial sections of IIM patients. (A-C) IBM: diffuse homogeneous MHC-II (A), diffuse MHC-I (B), and diffuse CD56 positivity (C). (D-F) IMNM: negative MHC-II (D), heterogeneous MHC-I (E), and diffuse CD56 positivity (F). (G-I) ASyS: perifascicular MHC-II (G), extended perifascicular MHC-I (H), and perifascicular CD56 positivity (I). (I-K) DM: negative MHC-II expression (J), diffuse MHC-I (K), and scattered CD56 positivity (L). (M-O) DM (patient with cancer-associated DM): perifascicular MHC-II (M), extended perifascicular MHC-I (N), and scattered CD56 positivity (O). (P-R) OM: heterogenous MHC-II (P), diffuse MHC-I (Q), and scattered CD56 positivity (R). Scale bars = 100 μm.

The MHC-II immunostaining patterns differed according to the IIM subgroup (Figures 3 and 4; Supplementary Data S2). IBM muscles showed MHC-II-positive myofibers in 100% of patients, mainly with diffuse immunostaining (*n* = 23/24, 96%). MHC-II myofiber immunostaining was positive in only 17% of IMNM patients (*n* = 3/18), the pattern of which was always scattered. Myofiber MHC-II expression was positive in 90% of the AsyS patients, with mainly perifascicular MHC-II immunostaining (*n* = 7/10, 70%). Among OM patients, 80% exhibited MHC-II myofiber expression, with either clustered (*n* = 8/20, 40%), perifascicular (*n* = 6/20, 30%), scattered (*n* = 4/20, 20%), or diffuse (*n* = 3/20, 15%) MHC-II positivity. In DM muscles, the expression of MHC-II by myofibers was inconsistent. MHC-II myofiber positivity was observed in 39% of the cases (*n* = 12/31), presenting as either perifascicular (*n* = 10/31, 32%), diffuse homogenous (1/31, 3%), or clustered (1/31, 3%).

Given the variability in MHC-II immunostaining observed among the DM muscles, we decided to further investigate these cases. Regarding the MSA status of DM patients, MHC-II myofiber positivity was found in 60% (*n* = 6/10) of anti-TIF1γ, 40% (*n* = 2/5) of anti-NXP2, 100% (*n* = 2/2) of anti-SAE, and 22% (2/9) of seronegative cases. No MHC-II myofiber positivity was detected in the three patients with anti-Mi2 nor the two patients with anti-MDA5. Regarding the characteristics of DM patients, 23% (*n* = 7/31) were juvenile and 23% (*n* = 7/31) presented with ongoing neoplasia at the time of DM diagnosis. When specifically considering these patients, 43% (*n* = 3/7) of the juvenile DM cases had a positive MHC-II immunostaining and 57% (*n* = 4/7) of DM with ongoing neoplasia displayed MHC-II positivity. Among the four DM patients with ongoing neoplasia and MHC-II myofiber positivity, two had lung adenocarcinoma and two had melanoma, while the three cases with negative MHC-II immunostaining were ovarian adenocarcinoma (*n* = 2) and ear neoplasia (*n* = 1; Table 3).

#### Differential MHC-I myofiber expression according to IIM subgroup

The patterns of MHC-I myofiber positivity differed according to the IIM subgroup (Figure 3; Supplementary Data S2). All IBM patients presented a diffuse MHC-I immunostaining, while in IMNM, a majority of patients displayed a scattered myofiber positivity (*n* = 13/18, 72%). In ASyS, myofiber MHC-I immunostaining was mainly perifascicular (*n* = 6/10, 60%) with either extended perifascicular (*n* = 4/10, 40%) or strictly perifascicular (*n* = 2/10, 20%) positivity. Myofiber MHC-I expression was positive in OM (*n* = 18/20, 90%), with mainly a diffuse pattern (*n* = 9/20, 45%) or a perifascicular pattern (*n* = 8/20, 40%). Finally, DM patients presented a diffuse homogenous pattern (*n* = 13/31, 42%) or a perifascicular pattern (*n* = 17/31, 55%). In addition, there was no obvious co-expression of MHC-I and MHC-II in DM, IMNM, and OM while in ASyS muscles both MHC-I and MHC-II immunostaining displayed a perifascicular pattern and in IBM muscles they displayed a diffuse pattern (Figures 2-4; Supplementary Data S2).

#### Myogenic status of myofibers expressing MHC-II

The co-expression of MHC-II and CD56 by myofibers was assessed using serial muscle sections for every tested IIM muscle biopsy (*n* = 98) to investigate the myogenic status of MHC-II-positive and -negative myofibers. The result could be analyzed in 92/98 biopsies and showed a co-expression in 60% of IIM patients (*n* = 55/92), which was apportioned as follows: 31% of DM (*n* = 9/29), 100% in IBM (*n* = 23/23), 23% of IMNM (*n* = 3/13), 100% of ASyS (*n* = 8/8), and 63% of OM (*n* = 12/19) (Figures 2-4; Supplementary Data S2). Of note, some muscle biopsies in which MHC-II immunostaining was negative had CD56-positive myofibers (DM *n* = 10/19, IMNM *n* = 15/15, OM *n* = 4/4).

Myofiber typing on serial muscle sections of IIM muscle biopsy with diffuse heterogeneous MHC-II myofiber immunostaining suggested that MHC-II-positive myofibers were mainly type II myofibers (Supplementary Data S3).

### Capillary immunohistochemistry study

#### Abnormal capillary MHC-II immunostaining in IIM muscle biopsies

The MHC-II muscle biopsy immunostaining also identified quantitative (capillary dropout, *n* = 47/88, 53%) and qualitative capillary abnormalities (defined by architectural abnormalities, including dilated and leaky capillaries, *n* = 79/98, 81%). Such qualitative impairments were frequently observed: leaky capillaries (*n* = 59/86, 67%) and dilated capillaries (*n* = 64/99, 65%) (Table 2; Figure 1; Supplementary Data S4).

#### Differential capillary MHC-II immunostaining according to IIM subgroup

**Figure 5.**
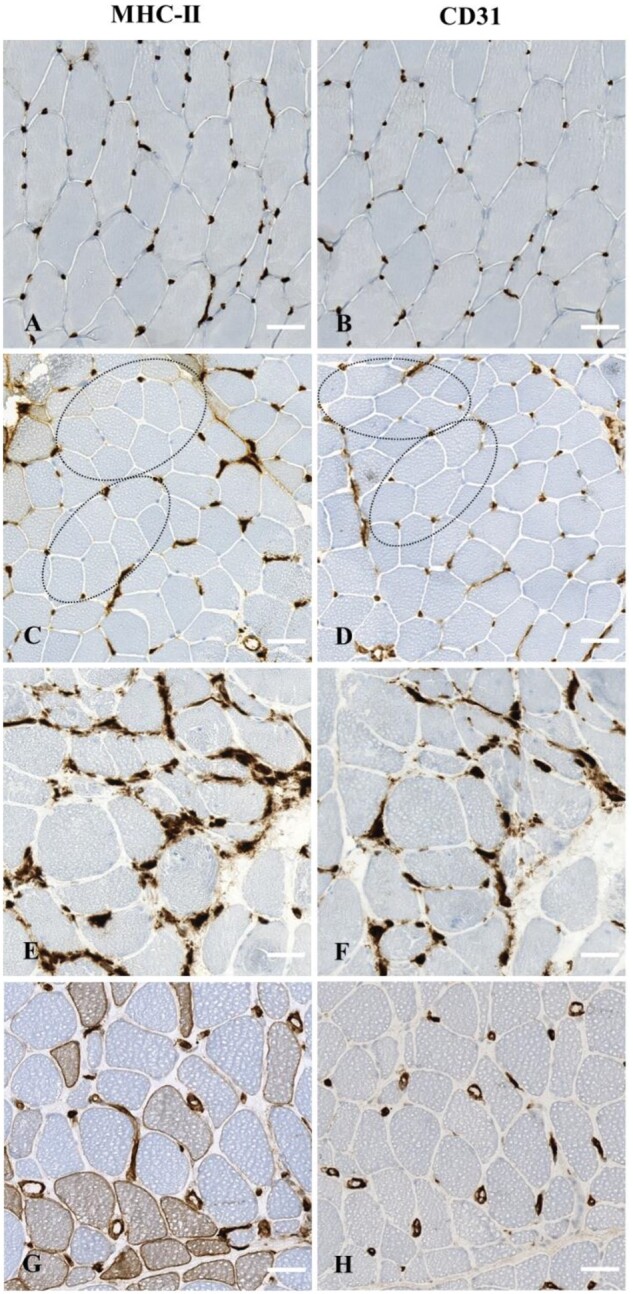
Illustrative images of major histocompatibility complex (MHC) class II and CD31 capillary immunostaining patterns on serial sections of muscle biopsies from controls and patients with idiopathic inflammatory myopathy. Normal MHC-II (A) and corresponding normal CD31 (B) immunostaining in a control patient. Capillary dropout observable upon MHC-II (C) and corresponding CD31 (D) immunostaining (dotted ellipses) in a patient with juvenile dermatomyositis. Leaky capillaries observable upon MHC-II (E) and corresponding CD31 (F) immunostaining in a patient with anti-synthetase syndrome. Dilated capillaries observable upon MHC-II (G) and corresponding CD31 (H) immunostaining in a patient with overlap myositis. Scale bars: 100 μm.

While almost all the IIM subgroups presented MHC-II quantitative and qualitative capillary abnormalities, some subgroups displayed specificities. MHC-II immunostaining identified that IBM muscles rarely presented capillary dropout (1/10, 10%) but frequently had dilated capillaries (*n* = 16/22, 73%). In IMNM, capillary dropout was reported in 39% of the cases and leaky and dilated capillaries were frequent (76% and 53%, respectively). In comparison, DM, ASyS, and OM appeared to have high frequencies of both quantitative and qualitative capillary abnormalities with the presence of capillary dropout, leaky capillaries, and dilated capillaries. Capillary CD31 immunostaining was evaluated for 98 IIM patients and identified capillary dropout (*n* = 58/98, 59%), leaky capillaries (54/95, 57%), and dilated capillaries (70/96, 73%). The concordance between the capillary abnormalities identified by MHC-II and CD31 immunostainings was high for both quantitative (capillary dropout, 85%) and qualitative impairments (leaky capillaries, 87% and dilated capillaries, 85%; Figures 3 and 5; Supplementary Data S4).

## Discussion

The present study identified the expression of MHC-II by myofibers as a potential diagnostic tool for IIM, providing elements for the distinction between IIM subgroups when considering the recent advances in IIM subgroup classification. The main patterns of myofiber MHC-II immunostaining found were: diffuse in IBM, perifascicular in ASyS, negative in IMNM, inconstantly positive in adult DM (mainly correlated with pediatric or neoplastic DM patients), and unspecific in OM (perifascicular, clustered, and diffuse heterogeneous). Second, the analysis of MHC-II capillary immunostaining of IIM muscles identified quantitative and qualitative capillary abnormalities in IIM, with high frequencies and specificities according to the different IIM subgroups.

### Myofiber MHC-II immunostaining, a tool for the diagnosis of IIM subgroups

As progress is made regarding IIM subgroup delineation, and since related care differs according to subgroups, the need for specific biomarkers becomes more apparent.^35^ Regarding MHC-II immunostaining in IIM muscle biopsies, most studies so far have evaluated MHC-II in polymyositis,^12^^,^^14^^,^^15^^,^^17^^,^^18^^,^^20^^,^^22–27^^,^^30^ an entity which is slowly disappearing as patients are re-classified mainly as ASyS, IMNM, OM, and DM-sine-dermatitis.^2^^,^^40^^,^^41^ Indeed, most previous studies were performed several decades ago, impeding the comparison with the currently used classification of IIM subgroups. Although some recent case studies report interesting results, each of them analyzed only a particular subgroup,^21^^,^^32–34^ thus impeding the identification of MHC-II myofiber immunostaining variation between IIM subgroups. The recent exhaustive and well-illustrated review by Nelke et al further highlights the usefulness of MHCI, MHCII, and complement immunostaining in IIM muscles.^42^ The present work illustrates the patterns proposed in this conceptual work by providing real-life data obtained from the routine histological examination of muscle biopsies during the IIM diagnostic process. Potential discrepancies between the above-mentioned study and the one herein will be further discussed.

Regarding IBM, the present study identified a mainly diffuse MHC-II myofiber immunostaining pattern, further strengthening and completing the results obtained in previous reports.^15^^,^^17^^,^^18^ IBM diagnosis is complex and many patients are currently facing diagnostic delays.^8^ The main reason for this is that IBM diagnosis is partly based on clinical features, notably distal muscle weakness, and on histological muscle lesions, notably rimmed vacuoles, which may appear late in the course of the disease.^2^ Achieving a diagnosis has important clinical implications since IBM is the only IIM that does not benefit from immunosuppressive therapy. In that context, MHC-II immunostaining, which seems to be detected early during the course of the disease, could be useful in reducing diagnostic delays.

Regarding the ASyS subgroup, the present results are in line with a recent study in which, among 33 ASyS and 17 DM patients, the authors identified a higher MHC-II positivity in ASyS compared to DM (81.8% vs 23.5%, respectively), and mainly with a perifascicular pattern.^21^ These results are strengthened by a recent study, that demonstrates MCH-II myofiber immunostaining positivity at both the mRNA and protein levels.^33^

Regarding IMNM, only few of the tested biopsies showed MHC-II-positive myofibers, a result in line with previously published cases.^16^^,^^43^ Another study, however, reported a higher frequency of positivity in IMNM (43.7%).^18^ This discrepancy could be explained by a difference in the diagnostic criteria applied, as herein none of the IMNM patients had anti-Jo1 or anti-PMScl Ab.

In the DM subgroup, as reported in the literature, MHC-II positivity was more variable than in the other subgroups. In a study including 14 DM, myofiber MHC-II-positive immunostaining was reported in 93% of cases,^17^ while in another study, MHC-II-positive myofibers were present in 54.5% of DM cases,^18^ a result closer to the one obtained herein. The lack of documented autoantibodies and the perifascicular immunostaining found in all cases raise the question of whether ASyS cases were not considered as DM in the first mentioned study.^17^ Interestingly, the recent study by Tanboon et al performed in an important cohort of 256 DM cases, found MHC-II positivity in only 18.1% of the cases and mainly in patients with anti-SAE and seronegative DM.^34^ In the recent review by Nelke et al, the authors report a correlation between MSA subtype and MHC-II myofiber immunostaining patterns, with negative staining in anti-Tif1γ, anti-MDA5, and anti-SAE, compared to sparsely perifascicular in anti-Mi2 and perifascicular in anti-NXP2.^42^ The present study, however, found MHC-II myofiber positivity in anti-Tif1γ, anti-NXP2, anti-SAE, and seronegative DM cases. These discrepancies reflect the complexity of IIM diagnosis and its underlying pathophysiological mechanisms, and call for caution during the interpretation of these various findings. Here, we noticed that the majority of DM patients with MHC-II-positive myofibers were either juvenile patients or patients with ongoing neoplasia. This is particularly interesting as Tanboon et al also reported that 40% of the anti-SAE DM cases were paraneoplastic and 85.7% of the seronegative DM cases concerned juvenile patients.^34^ Although this observation requires further investigation on larger groups of patients, the use of MHC-II immunostaining could be of great value for adult DM patients, as it could represent a new tool, easily usable in routine, to detect a paraneoplastic process. Regarding juvenile cases, in line with the present results, a recent study identified a positive MHC-II myofiber immunostaining in 7 out of 9 cases of juvenile DM, with a mild intensity and a scattered distribution, except for 2 cases which showed a perifascicular pattern.^32^ Another study, in 2009, found MHC-II-positive myofiber immunostaining in only 28% of juvenile DM muscles.^30^ Since that time however, progresses have been made in juvenile DM diagnostic criteria. Moreover, a large number of patients from that study had received treatment prior to their biopsy, and the age cut-off for defining juvenile DM differed from the study herein.

With regards to OM, the very variable patterns of MHC-II myofiber positivity may reflect the lack of specificity of this IIM subgroup and argue for the need to further refine their classification. Overall, the present results show that MHC-II immunostaining could be a useful additional tool for IIM diagnosis, particularly for distinguishing IIM subgroups.

### Input for the understanding of IIM pathogenesis

The immunostainings carried out on serial sections allowed us to identify that a non-negligible proportion of myofibers expressing MHC-II are regenerating myofibers (CD56-positive). While the fact that myofibers in IIM damaged muscles undergo regeneration appears logical, the expression of MHCII is unexpected. MHC-II molecules are known to play a pivotal role in the induction and regulation of immune responses through their ability to present antigens to CD4+ T lymphocytes. The expression of MHC-II by myofibers in some IIM subgroups thus questions the potential immune role of myofibers. Indeed, while the expression of MHC-II molecules is constitutive of APCs (monocytes/macrophages, B cells, and dendritic cells), this expression appears to be inducible in most cell types and tissues under specific inflammatory conditions.^44^ In the present work, the fact that myofibers express MHC-II raises the question of myofibers acting as potential APCs. Some studies have demonstrated the potential role of myogenic cells as APCs.^13^^,^^45^ Another study has demonstrated that, for some IIM patients, the myofibers expressing MHC-II also express the intracellular adhesion molecule-1, a molecule required for the stabilization of the immunological synapse between MHC-peptide complex and T cells.^24^ These considerations advocate for an active role of myofibers in the complex dysimmune cascade that underlies IIM pathogenesis.

Another interesting point is the observation that type II myofibers (identified thanks to multiple techniques) were the myofibers that preferentially expressed MHC-II in case of heterogeneous positivity. This finding raises the hypothesis of a potential link between the glycolytic/oxidative metabolism of myofibers and their involvement in the immune process. Also, this observation is in line with the atrophy of type II myofibers reported in myopathy associated with systemic inflammatory disorders.^46^

Concerning underlying mechanisms, IFN-γ has been identified as a strong inducer of MHC-II cellular expression.^44^ Interestingly, a recent study demonstrated that IBM muscles display a strong IFN type II (ie, IFN-γ) signature which is not the case of other IIM.^47^ In this context, the mainly diffuse expression of MHC-II observed in IBM myofibers is in line with such a mechanism.

The identification of distinct patterns of MHC-II myofiber immunostaining among IIM subgroups also confirms the progress made in delineating this group of diseases and advocates for pathogenic-specific processes among these subgroups. For example, the mainly perifascicular MHC-II myofiber immunostaining found in ASyS strengthens the consideration of a specific disease, individualized from the OM subgroup. Regarding DM, the present findings support the idea of potentially distinct pathogenic processes between juvenile and adult DM, which has been previously discussed in another study.^32^ Indeed, while sharing pathognomonic cutaneous lesions and muscle inflammation, some clinical features differ between adult and pediatric cases, notably the TIF1γ -associated malignancy and anti-MDA5-associated rapidly progressive interstitial lung disease, which are found only in adult DM.^48^ Moreover, in the adult paraneoplastic DM cases, the MHC-II negative cases were associated with ongoing ovarian adenocarcinoma, while those that showed MHC-II-positive myofiber immunostaining were melanoma and bronchial adenocarcinoma cases. Altogether, this questions the hormonal implication during DM pathogenesis, and these results call for larger studies.

### Capillary impairment, a frequent element in all IIM subgroups

The present study identified abnormal muscle capillary immunostaining (by both MHC-II and CD31) as a common feature of IIM. The observed microvasculature changes observed were both quantitative and qualitative impairments and found in various proportions in the different IIM subgroups. While capillary dropout appeared practically absent from IBM and IMNM muscles, qualitative abnormalities appeared to be largely shared by all IIM subgroups, a result in line with the only other study having evaluated capillary impairment in the currently defined IIM subgroups.^39^ When assessing in more detail the types of abnormalities present, the current study identified both dilated and leaky capillaries. While dilated capillaries have rarely been described,^39^ these could resemble the pipestem capillaries reported by several other studies. These are defined by a thickening of the capillary walls, characterized by the absence of undulating tubules and the presence of an amorphous material other than amyloid.^49–51^ Of note, no association was found between diabetes and the occurrence of capillary dropout or dilated capillaries in the present study (data not shown). To our knowledge, leaky capillaries have never been reported in IIM. The observation of these capillary abnormalities using both MHC-II and CD31 immunostaining argues for a non-artefactual result. In such capillaries, the sprout aspect emanating from endothelial cells resembles that described when pre-existing capillaries incorporate resident or circulating endothelial progenitor cells.^52^ This result needs to be confirmed on larger cohorts and should be further investigated. In terms of diagnosis, CD31 immunostaining appeared more effective to detect capillary dropout and dilated capillaries, while MHC-II immunostaining identified more effectively leaky capillary lesions.

To date, microvasculature changes in IIM have been mainly described in DM cases, and these include endothelial inclusion,^39^^,^^53–55^ capillary depletion,^56–58^ and a significant increase in neovascularization, particularly in juvenile DM.^59^ Interestingly, vasculopathy has been significantly linked to muscle damage^60^ and to the severity of the disease, in both juvenile and adult DM.^56^^,^^61^ In line with the present findings, a recent study using ultrastructural analysis on 60 patients identified that capillary dropout was mainly present in DM, ASyS, and scleromyositis (an entity included in the OM subgroup).^39^ Regarding the OM subgroup, two recent studies identified basal membrane thickening and reduplications, endothelial activation, and pericyte proliferation in scleromyositis.^39^^,^^62^ One study using ultrastructural analysis reported a thickening of basal membrane in IMNM and basal membrane reduplication, increased number of pericyte processes, and endothelial activation in IBM,^39^ while another study reported that the microvascular architecture in IBM was distorted but without providing details.^63^ Considering the potential pathogenic processes at play, one study demonstrated that polymyositis and DM were associated with phenotypic and functional dysregulation of endothelial precursor cells, which may be related to IL-18 and IFN-I.^64^

## Limitations and perspectives

Although carried out on a retrospective series, the present work allowed to evaluate MHC-II immunostaining in the five well-defined IIM subgroups. The blind pathological analysis was performed by four trained muscle pathologists, allowing an unbiased histological interpretation. However, the analysis of additional cases would help further support the inclusion of MHC-II immunostaining in routine histological assessment of IIM muscle biopsies. The qualitative capillary impairments identified by MHC-II and CD31 immunostaining call for further investigation, notably by including an ultrastructural analysis.

## Conclusion

The present work, carried out on a cohort of patients representative of the currently established IIM subgroups, demonstrates that MHC-II expression allows to distinguish between several subgroups, thanks to pattern specificity: MHC-II staining is diffuse in IBM, negative or sparse in IMNM, perifascicular in ASyS, but variable in DM and OM. Such a readily available and early marker could be useful as a complement to the currently used histological tools for IIM diagnosis and classification. Further investigations regarding capillary alterations and pathogenic mechanisms in IIM are required.

## Supplementary Material

nlae098_Supplementary_Data

## Data Availability

Anonymized data can be made available upon reasonable request to the corresponding author.
