# Supplementary material for: Contribution of major histocompatibility complex class II immunostaining in distinguishing idiopathic inflammatory myopathy subgroups: A histopathological cohort study
Source: J Neuropathol Exp Neurol. 2024 Sep 16;83(12):1060–75. doi: 10.1093/jnen/nlae098 (PMC11576552; doi:10.1093/jnen/nlae098)
Supplement: nlae098_Supplementary_Data [file nlae098_supplementary_data.zip › nlae098_Supplementary_Data/Rays edited Supplemental Data 1. Ab references 1.7.24.docx]

**Supplemental Information 1. Monoclonal antibody panel used for the immunohistochemistry analysis.**

|  | **Marker for** | **Antibody Dilution** | **Reference and supplier** |
| --- | --- | --- | --- |
| anti-MHC-I | HLA-ABC | 1/2400 | DAKO ref: M0736, mouse monoclonal (clone W6/32), HLA class I histocompatibility antigen |
| anti-MHC-II | HLA-DP beta 1 chain, DQ beta 1 chain, and DRB1 beta chain | 1/400 | DAKO ref: M0775, mouse monoclonal (clone CR3/43), HLA class II histocompatibility antigen |
| anti-CD31 | Vascular endothelial cells | 1/50 | Ventana ref: 760-4378, prediluted, mouse monoclonal (clone JC70A), CD31 |
| anti-CD56 | Regenerating myofibers (NCAM+) | 1/50 | Cell Marque ref: 156-M85, mouse monoclonal, (clone 123C3), Human CD56 antigen |

In all experiments, an isotype-matched irrelevant antibody (DAKO, Roche, Leica) was included as negative control. HLA: human leucocyte antigen; MHC: major histocompatibility complex.
