# Supplementary material for: Contribution of major histocompatibility complex class II immunostaining in distinguishing idiopathic inflammatory myopathy subgroups: A histopathological cohort study
Source: J Neuropathol Exp Neurol. 2024 Sep 16;83(12):1060–75. doi: 10.1093/jnen/nlae098 (PMC11576552; doi:10.1093/jnen/nlae098)
Supplement: nlae098_Supplementary_Data [file nlae098_supplementary_data.zip › nlae098_Supplementary_Data/Rays edited Supplemental Data 2.docx]

**Supplemental Data 2. Major histocompatibility complex (MHC) class II, I, and CD56 myofiber immunostaining in IIM patients.**

|  | **DM** | **IBM** | **IMNM** | **ASyS** | **OM** | **total** |
| --- | --- | --- | --- | --- | --- | --- |
| Patients (n) | 31 | 24 | 18 | 10 | 20 | 103 |
| **MHC-II patterns** | | | | | | |
| MHC-II positive myofibers | 12/31 (39%) | 24/24 (100%) | 3/18 (17%) | 9/10 (90%) | 16/20 (80%) | 63/103 (61%) |
| Diffuse MHC-II expression | 1/31 (3%) | 23/24 (96%) | 0 | 1/10 (10%) | 3/20 (15%) | 28/103 (28%) |
| - homogenous | 1/31 (3%) | 7/24 (29%) | 0 | 0 | 0 | 8/103 (8%) |
| - heterogenous | 0 | 16/24 (67%) | 0 | 1 (10%) | 3/20 (15%) | 20/103 (19%) |
| Perifascicular MHC-II expression | 10/31 (32%) | 0 | 0 | 7/10 (70%) | 6/20 (30%) | 23/103 (22%) |
| - strictly perifascicular | 9/31 (29%) | 0 | 0 | 5/10 (50%) | 5/20 (25%) | 19/103 (18%) |
| - extended perifascicular | 1/31 (3%) | 0 | 0 | 2/10 (20%) | 1/20 (5%) | 4/103 (4%) |
| Scattered MCH-II expression | 0 | 0 | 3/18 (17%) | 1/10 (10%) | 4/20 (20%) | 8/103 (8%) |
| Cluster MHC-II expression | 1/31 (3%) | 1/24 (4%) | 0 | 2/10 (20%) | 8/20 (40%) | 12/103 (12%) |
| MHC-II negative myofibers | 19/31 (61%) | 0 | 15/18 (83%) | 1/10 (10%) | 4/20 (20%) | 39/103 (38%) |
| **MHC-I patterns** | | | | | | |
| MHC-I positive | 30/31 (97%) | 24/24 (100%) | 16/18 (89%) | 10/10 (100%) | 18/20 (90%) | 98/103 (95%) |
| Diffuse MHC-I expression | 13/31 (42%) | 24/24 (100%) | 3/18 (17%) | 2/10 (20%) | 9/20 (45%) | 51/103 (50%) |
| - homogenous | 13/31 (42%) | 17/24 (71%) | 0 | 2/10 (20%) | 7/20 (35%) | 39/103 (38%) |
| - heterogenous | 0 | 7/24 (29%) | 3/18 (17%) | 0 | 2/20 (10%) | 12/103 (12%) |
| Perifascicular MHC-I | 17/31 (55%) | 0 | 0 | 6/10 (60%) | 8/20 (40%) | 31/103 (30%) |
| - strictly perifascicular | 6/31 (19%) | 0 | 0 | 2/10 (20%) | 3/20 (15%) | 11/103 (11%) |
| - extended perifascicular | 11/31 (35%) | 0 | 0 | 4/10 (40%) | 5/20 (25%) | 20/103 (19%) |
| Scattered MHC-I expression | 1/31 (3%) | 0 | 13/18 (72%) | 2/10 (20%) | 1/20 (5%) | 17/103 (17%) |
| MHC-I negative myofibers | 1/31 (3%) | 0 | 2/18 (11%) | 0 | 2/20 (10%) | 5/103 (5%) |
| Co-staining with MHC-II | 5/31 (16%) | 21/24 (88%) | 3/18 (17%) | 6/10 (60%) | 11/20 (55%) | 46/103 (45%) |
| **CD56** | | | | | | |
| CD56 positive myofibers | 29/29 (100%) | 24/24 (100%) | 18/18 (100%) | 8/8 (100%) | 18/19 (95%) | 97/98 (99%) |
| Co-staining with MHC-II | 9/29 (31%) | 23/23 (100%) | 3/13 (23%) | 8/8 (100%) | 12/19 (63%) | 55/92* (60%) |
| Co-staining in MHC-II positive cases | 8/11 (73%) | 23/23 (100%) | 3/3 (100%) | 7/7 (100%) | 12/19 (63%) | 53/63 (84%) |

*** For 6 cases serial sections did not allow myofiber correspondence.**
