# Supplementary material for: Contribution of major histocompatibility complex class II immunostaining in distinguishing idiopathic inflammatory myopathy subgroups: A histopathological cohort study
Source: J Neuropathol Exp Neurol. 2024 Sep 16;83(12):1060–75. doi: 10.1093/jnen/nlae098 (PMC11576552; doi:10.1093/jnen/nlae098)
Supplement: nlae098_Supplementary_Data [file nlae098_supplementary_data.zip › nlae098_Supplementary_Data/Rays edited Supplemental Data 3. Myosin heavy chain.docx]

**Supporting Information 3. Fiber typing in muscle biopsies from IIM patients muscles presenting a diffuse heterogeneous major histocompatibility complex (MHC) class II myofibers expression.**


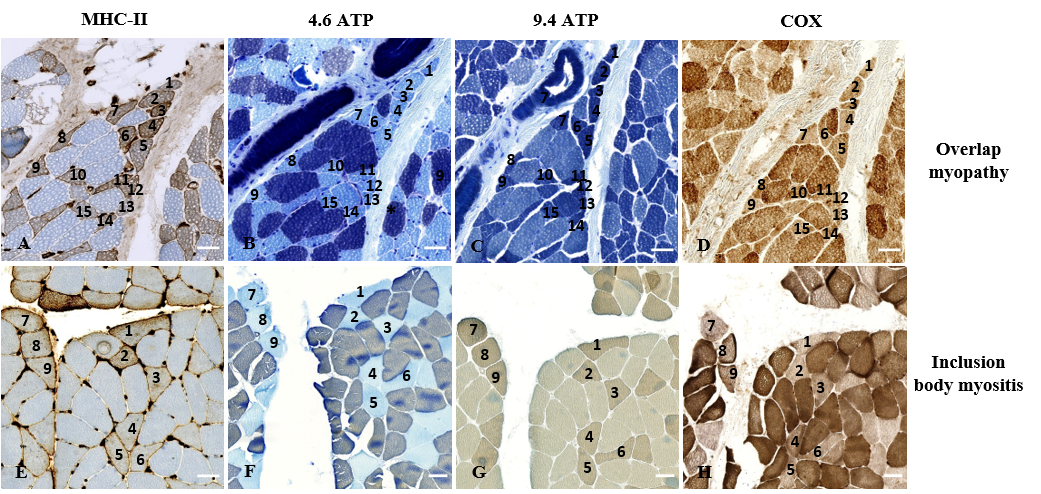


MHC-II immunostaining is shown in (A,E). Myosin adenosine triphosphatase staining was performed either in acidic condition (pH=4.6) highlighting dark type I and light type II myofibers, or in alkaline condition (pH=9.4) highlighting light type I and dark type II myofibers, with or without blue toluidin treatment. Histoenzymatic staining for the activity of the cytochrome c oxidase (COX, complex V of the mitochondrial respiratory chain) highlights type I myofibers in dark brown, and type II myofibers in light brown (D,H). A given number shows the same myofiber in serial sections. (A-D) In OM muscle, 15 MHC-II positive myofibers (A), were type II myofibers (light in B, dark in C and low COX activity in D). (E-H) In IBM muscle, 9 MHCI-II positive myofibers (E), were type II myofibers (light in F, dark in G and low COX activity in H).

Scale bars: 50 μm.
