# Supplementary material for: Contribution of major histocompatibility complex class II immunostaining in distinguishing idiopathic inflammatory myopathy subgroups: A histopathological cohort study
Source: J Neuropathol Exp Neurol. 2024 Sep 16;83(12):1060–75. doi: 10.1093/jnen/nlae098 (PMC11576552; doi:10.1093/jnen/nlae098)
Supplement: nlae098_Supplementary_Data [file nlae098_supplementary_data.zip › nlae098_Supplementary_Data/Rays edited Supplemental Data 4. Capillaries .docx]

**Supplemental Data 4. MHC-II and CD31 capillary immunostaining in IIM patients**

|  | DM | IBM | IMNM | ASyS | OM | total |
| --- | --- | --- | --- | --- | --- | --- |
| Patients (n) | 31 | 24 | 18 | 10 | 20 | 103 |
| **Capillary quantitative abnormality: capillary dropout** | | | | | | |
| MHC-II capillary dropout | 21/31 (68%) | **1/10 (10%)*** | 7/18 (39%) | 4/9 (44%) | 14/20 (70%) | 47/88 (53%) |
| CD31 capillary dropout | **24/30 (80%)** | 2/23 (9%) | **11/17 (65%)** | **6/10 (60%)** | **15/18 (83%)** | 58/98 (59%) |
| *Correlation of CD31 with MHC-II* | *26/30 (87%)* | *10/10 (100%)* | *13/17 (76%)* | *7/9 (78%)* | *15/18 (83%)* | *71/84 (85%)* |
| **Capillary qualitative abnormalities: leaky or dilated capillaries** | | | | | | |
| MHC-II capillary structural abnormalities | 22/31 (68%) | 17/21 (81%) | 15/17 (88%) | 10/10 (100%) | 15/19 (79%) | 79/98 (81%) |
| Leaky MHC-II capillaries | 22/31 (71%) | **5/10 (50%)*** | **13/17 (76%)** | **6/9 (67%)** | **13/19 (68%)** | 59/86 (67%) |
| Leaky CD31 capillaries | **24/30 (80%)** | 8/22 (36%) | 9/16 (56%) | 5/10 (50%) | 8/17 (47%) | 54/95 (57%) |
| *Correlation of CD31 with MHC-II* | *26/30 (87%)* | *10/10 (100%)* | *13/16 (81%)* | *8/9 (89%)* | *14/17 (82%)* | *71/82 (87%)* |
| Dilated MHC-II capillaries | 21/31 (68%) | 16/22 (73%) | 9/17 (53%) | 9/10 (90%) | 9/19 (47%) | 64/99 (65%) |
| Dilated CD31 capillaries | **23/30 (77%)** | **17/23 (74%)** | **9/16 (56%)** | **9/10 (90%)** | **12/17 (71%)** | 70/96 (73%) |
| *Correlation of CD31 with MHC-II* | *23/30 (77%)* | *17/21 (81%)* | *16/16 (100%)* | *9/9 (100%)* | *14/17 (82%)* | *79/93 (85%)* |

Values in bold are the highest value of an elementary lesion (i.e. capillary dropout, leaky, or dilated capillaries); values in red are the highest value for each IIM subgroup. *Difficult to assess due to diffuse myofiber positivity.
